# Molecular Profiling of CRISPR‐Cas System, Virulence Traits, and Antimicrobial Resistance in *Enterococcus faecalis* Clinical Isolates

**DOI:** 10.1155/ijm/5903066

**Published:** 2026-07-26

**Authors:** Moein Nikravan, Hamid Heidari, Saeed Khoshnood, Sobhan Ghafourian, Hossein Kazemian

**Affiliations:** ^1^ Department of Microbiology, Faculty of Medicine, Ilam University of Medical Sciences, Ilam, Iran, medilam.ac.ir; ^2^ Department of Microbiology, Faculty of Medicine, Shahid Sadoughi University of Medical Sciences, Yazd, Iran, ssu.ac.ir; ^3^ Clinical Microbiology Research Center, Ilam University of Medical Sciences, Ilam, Iran, medilam.ac.ir

**Keywords:** CRISPR-Cas, *Enterococcus faecalis*, ERIC-PCR, high-level gentamicin resistant (HLGR), vancomycin-resistant enterococci (VRE)

## Abstract

**Introduction:**

*Enterococcus faecalis* is responsible for life‐threatening enterococcal infections. This study is aimed at investigating virulence factors, antimicrobial resistance patterns, and molecular characteristics of clinical *E. faecalis* isolates.

**Materials and Methods:**

A total of 42 *E. faecalis* isolates were collected. Antimicrobial resistance and the minimum inhibitory concentrations (MICs) of vancomycin and gentamicin were determined using standard microbroth dilution method. The presence of virulence, antibiotic resistance, and CRISPR‐Cas genes was investigated by polymerase chain reaction (PCR). Biofilm formation was also assessed, and the genetic diversity of the isolates was analyzed using enterobacterial repetitive intergenic consensus‐polymerase chain reaction (ERIC‐PCR).

**Results:**

The highest resistance rates were observed for gentamicin and ampicillin, and all isolates were high‐level gentamicin resistant (HLGR) (MICs ≥ 500 *μ*g/mL). Vancomycin resistance was detected in 15 isolates (35.7%) (MICs ≥ 32 *μ*g/mL). Thirty‐nine isolates (92.8%) were biofilm producers. The *efaA* gene was the most frequently detected (95.2%), followed by *esp* (69%), *gelE* (66.6%), *ace* (66.6%), and *asa1* (57.1%). The *aac*(*6*  ^′^)*-Ie-aph*(*2*  ^″^)*-Ia* gene was detected in all isolates (100%), followed by *ermB* (78.6%), *ermA* (38.1%), *ermC* (23.8%), and *vanA* (19%). CRISPR3 was the most frequently detected locus (80.95%), followed by CRISPR1 (50%), CRISPR1‐cas *csn1* (23.8%), and CRISPR2 (9.5%). The CRISPR3‐cas *csn1* gene was not detected in any isolate. High heterogeneity was observed among the isolates, with 35 different ERIC types identified.

**Conclusion:**

This study demonstrated notable resistance traits and high genetic diversity among clinical *E. faecalis* isolates, but no statistically significant association was found between CRISPR‐Cas genes and phenotypic or genotypic resistance.

## 1. Introduction

Enterococcal species are gram‐positive bacteria that primarily inhabit the throat, mouth, intestines, and vagina, where they exist as commensal organisms. These organisms were formerly thought to have minimal pathogenic potential, but it is now recognized that they can cause severe infections, especially bacteremia and endocarditis [1, 2]. They are also involved in various infections, including urinary tract, intra‐abdominal, wound, and catheter‐associated infections, as well as suppurative thrombophlebitis and endocarditis [3]. Enterococci rank as the second most common causative agents of hospital‐associated infections (HAIs), contributing significantly to morbidity and mortality [4]. *Enterococcus faecalis* and *Enterococcus faecium* are the most prevalent pathogens in healthcare settings worldwide, with *E. faecalis* being the more common and virulent species, responsible for serious high‐inoculum infections such as infective endocarditis [3, 4].

Multidrug resistance is a rising public health issue, largely because of the potential ineffectiveness of treatment for enterococcal infections, particularly among immunocompromised patients. Multidrug‐resistant (MDR) bacterial species can develop patterns of antibiotic resistance, leading to severe hospital‐acquired infections [5]. These microorganisms also exhibit intrinsic resistance to various classes of antimicrobials, including cephalosporins, aminoglycosides, lincosamides, and trimethoprim–sulfamethoxazole [5]. Furthermore, they can acquire resistance to numerous antimicrobial agents, such as aminoglycosides (high‐level gentamicin resistance [HLGR]), vancomycin (vancomycin‐resistant enterococci [VRE]), tetracyclines, and macrolides [4, 6]. In enterococci, glycopeptide resistance is facilitated by different mobile gene clusters, including *vanA*, *vanB*, *vanC*, *vanD*, *vanE*, *vanG*, *vanL*, *vanM*, and *vanN* [7].

The *vanA* genotype represents the predominant form of enterococcal vancomycin resistance in various countries [8]. The main mechanism underlying aminoglycoside resistance involves the acquisition of resistance genes that encode different aminoglycoside‐modifying enzymes (AMEs). This results in significant resistance to aminoglycosides, with the most clinically important gene being the bifunctional *aac*(*6*  ^′^)*-Ie–aph*(*2*  ^″^)*-Ia* gene [9, 10]. Additionally, virulence factors contribute to bacterial attachment to host cells or extracellular matrix (ECM) proteins and play a role in evading the immune response [11]. In particular, the production of cell surface proteins, the ability to maintain cell envelope integrity, adaptation to available nutrient sources, and the potential for biofilm formation substantially enhance gastrointestinal (GI) colonization and have important implications for horizontal gene transfer [12]. Research has linked enterococcal virulence to several factors, including enterococcal surface protein (Esp), aggregation substance (Asa1), and gelatinase (GelE), which enhance adherence to renal tubular cells and promote biofilm formation [13]. Cytolysin (*CylA*) is responsible for the lysis of blood cells, whereas hyaluronidase (*Hyl*) assists in the colonization of host tissues. Moreover, *Esp* contributes to increased adherence and colonization by enterococci. Enterococci are recognized for their ability to transfer antibiotic resistance to other bacterial species through mobile genetic elements, particularly plasmids and transposons. They play a key role in the spread and acquisition of antibiotic resistance genes, frequently exchanging these genes between virulent and resistant strains [1, 5].

One factor that may limit the emergence and spread of antibiotic resistance in bacteria is the clustered regularly interspaced short palindromic repeat (CRISPR)‐Cas system [14]. These systems act as defense mechanisms in prokaryotes and play a vital role in adaptive immunity by targeting genetic elements. Antibiotic selection influences the outcome of this interaction by favoring CRISPR mutants or the deletion of genes targeted by CRISPR [14, 15]. The CRISPR‐Cas mechanism comprises three stages: adaptation, expression, and interference. Genomic studies have demonstrated interactions between CRISPR‐Cas systems and mobile genetic elements. *E. faecalis* possesses a Type II CRISPR‐Cas system. Its genome contains three CRISPR loci, namely CRISPR1‐Cas, CRISPR2, and CRISPR3‐Cas [14, 16, 17]. Although research worldwide has consistently shown an inverse relationship between CRISPR‐Cas immune systems and multidrug resistance [16, 17], local antibiotic usage often shapes genomic patterns in unexpected ways. In Iran, the significant clinical prevalence of VRE and high‐level aminoglycoside resistance (HLAR) makes it essential to examine our own unique strain profiles. We designed the present study to evaluate the CRISPR‐mediated defense system in *E. faecalis* in the context of local antibiotic pressures. Therefore, the aim of this study was to examine virulence traits, antimicrobial resistance, CRISPR patterns, and molecular relatedness among clinical *E. faecalis* isolates.

## 2. Materials and Methods

### 2.1. Study Population and Bacterial Isolates

This cross‐sectional study was conducted between October 2021 and July 2022. The study protocol was approved by the Ethics Committee of Ilam University of Medical Sciences (IR.MEDILAM.REC.1402.091). Bacterial isolates were collected from two teaching hospitals in Ilam (Imam Khomeini and Mustafa Khomeini). The samples were obtained from urinary tract infections (*n* = 19), wounds (*n* = 12), and bloodstream infections (*n* = 11). Isolates were identified to the species level based on colony morphology, Gram staining, the catalase test, bile‐esculin hydrolysis, growth in brain–heart infusion broth containing 6.5% sodium chloride, and the production of acid from lactose, mannitol, and arabinose, as well as arginine hydrolysis and pyruvate fermentation [18]. Identification to the species level was confirmed via polymerase chain reaction (PCR) using specific primers targeting the *ddl–E. faecalis* gene (Table 1) [19]. *E. faecalis* ATCC29212 was used as reference strain (positive control).

**Table 1:** The list of primers used in this study.

Gene	Sequence (5 ^′^–3 ^′^)	Amplicon size (b.p.)	Ref.
*ddl–E. faecalis*	F:ATCAAGTACAGTTAGTCT	941	[19]
	R:ACGATTCAAAGCTAACTG		
*esp*	F:TTGCTAATGCTAGTCCACGACC	954	[5]
	R:GCGTCAACACTTGCATTGCCGAA		
*gelE*	F:ACCCCGTATCATTGGTTT	419	[5]
	R:ACGCATTGCTTTTCCATC		
*cylA*	F:TGGATGATAGTGATAGGAAGT	517	[5]
	R:TCTACAGTAAATCTTTCGTCA		
*hyl*	F:ACAGAAGAGCTGCAGGAAATG	276	[5]
	R:GACTGACGTCCAAGTTTCCAA		
*asa1*	F:GGTGCCACAATCAAATTAGG	380	[5]
	R:GATTCTTCGATTGTGTTGTAAACG		
*ace*	F:GGAATGACCGAGAACGATGGC	617	[5]
	R:GCTTGATGTTGGCCTGCTTCCG		
*efaA*	F:CGTGAGAAAGAAATGGAGGA	500	[5]
	R:CTACTAACACGTCACGAATG		
*vanA*	F:AATACTGTTTGGGGGTTGCTC	732	[19]
	R:CTTTTTCCGGCTCGACTTCCT		
*vanB*	F:CATCGCCGTCCCCGAATTTCAAA	635	[19]
	R:GATGCGGAAGATACCGTGGCT		
*aac*(*6* ^′^)*-Ie-aph*(*2* ^″^)*-Ia*	F:CAGGAATTTATCGAAAATGGTAGAAAAG	369	[20]
	R:CACAATCGACTAAAGAGTACCAATC		
*ermA*	F:TATCTTATCGTTGAGAAGGGATT	139	[21]
	R:CTACACTTGGCTTAGGATGAAA		
*ermB*	F:CTATCTGATTGTTGAAGAAGGATT	142	[21]
	R:GTTTACTCTTGGTTTAGGATGAAA		
*ermC*	F:CTTGTTGATCACGATAATTTCC	190	[21]
	R:ATCTTTTAGCAAACCCGTATTC		
*msrA*	F:TCCAATCATTGCACAAAATC	163	[21]
	R:AATTCCCTCTATTTGGTGGT		
CRISPR1‐*cas csn1*	CAGAAGACTATCAGTTGGTG	783	[16]
	CCTTCTAAATCTTCTTCATAG		
CRISPR1‐*cas* loci	GCGATGTTAGCTGATACAAC	315	[16]
	CGAATATGCCTGTGGTGAAA		
CRISPR2 loci	CTGGCTCGCTGTTACAGCT	Variable	[16]
	GCCAATGTTACAATATCAAACA		
CRISPR3‐*cas csn1*	GCTGAATCTGTGAAGTTACTC	258	[16]
	CTGTTTTGTTCACCGTTGGAT		
CRISPR3‐*cas* loci	GATCACTAGGTTCAGTTATTTC	224	[16]
	CATCGATTCATTATTCCTCCAA		

### 2.2. Antimicrobial Susceptibility Testing

Antibacterial susceptibility patterns were assessed using the disk diffusion method with tetracycline (30 *μ*g), nitrofurantoin (300 *μ*g), ampicillin (10 *μ*g), linezolid (30 *μ*g), tigecycline (15 *μ*g), chloramphenicol (30 *μ*g), and ciprofloxacin (5 *μ*g). The zone of inhibition was measured according to the standards established by CLSI recommendations [22]. The minimum inhibitory concentration (MIC) of vancomycin was determined by broth microdilution. In addition, the microdilution method was used to screen for HLGR strains (MIC ≥ 500 *μ*g/mL). The results were interpreted according to CLSI guidelines. *E. faecalis* ATCC 29212 and *Staphylococcus aureus* ATCC 25923 were used as quality control strains [22].

### 2.3. Biofilm Formation Assay

The capacity of *Enterococcus* isolates to form biofilms was assessed using a standardized microtiter plate assay [5]. In brief, *Enterococcus* isolates were inoculated into 5 mL of trypticase soy broth (TSB) supplemented with 0.5% glucose and incubated overnight at 37°C. A 0.5 McFarland standard concentration was then prepared in TSB. Subsequently, 100 *μ*L of these standardized suspensions were introduced into each well of a 96‐well, flat‐bottomed polystyrene microtiter plate. After a 24‐h incubation period at 37°C, the culture supernatants were carefully removed. The wells were then subjected to three sequential washes with 150 *μ*L of sterile normal saline (0.9% NaCl) to eliminate planktonic cells. To fix the adherent biofilm biomass, 96% ethanol was applied to the wells. Following air‐drying of the fixed biofilms, the plates were stained with 100 *μ*L of a 1.5% aqueous solution of crystal violet for 20 min. Excess stain was removed through three washes with distilled water. The bound crystal violet was then solubilized using 150 *μ*L of 33% (v/v) acetic acid. The resulting optical densities (ODs) were measured at a wavelength of 550 nm using a microplate reader. Each isolate was tested in triplicate. *S. epidermidis* ATCC 35984 and sterile TSB served as the positive and negative controls, respectively. A cutoff value (ODc) was established, defined as three standard deviations (SD) above the mean OD of the negative control: ODC = average OD of the negative control + (3 × SD of the negative control). The isolates were categorized into the following four groups based on the OD: nonbiofilm producer (OD < ODc); weak‐biofilm producer (ODc < OD < 2 × ODc); moderate‐biofilm producer (2 × ODc < OD < 4 × ODc); and strong‐biofilm producer (OD ≥ 4 × ODc).

### 2.4. Molecular Detection of Virulence Factor, Antibiotic Resistance, and CRISPR‐Cas Genes

Genomic DNA was extracted from fresh, pure colonies of isolated *E. faecalis* using the boiling method [23]. In brief, several colonies were suspended in sterile distilled water and heated in a dry bath at 95°C for 15 min. The samples were then centrifuged at 13,000 rpm for 10 min. The resulting supernatant was used as the DNA template. The extracted DNA was stored at −20°C until use. DNA quality was assessed by measuring the absorbance ratio at 260/280 nm using a NanoDrop spectrophotometer [24]. PCR was performed to detect genes encoding virulence factors (*gelE*, *esp*, *cylA*, *hyl*, *asa1*, *ace*, and *efaA*) [5], antibiotic resistance genes, including those for glycopeptides (*vanA* and *vanB*) [19], macrolides (*ermA*, *ermB*, *ermC*, and *msrA*) [21], aminoglycosides (*aac*(*6*  ^′^)*-Ie-aph*(*2*  ^″^)*-Ia*) [20], as well as CRISPR‐Cas genes [16], using the specific primers listed in Table 1. Finally, PCR products were analyzed by electrophoresis on 1% agarose gel, followed by staining with stain load dye (CinnaGen Co., Iran). *E. faecalis* ATCC 29212 and previously confirmed gene‐positive control strains were used as positive reference controls for targeted PCR amplification, whereas sterile master mix without a DNA template served as the negative control in each PCR run.

### 2.5. Enterobacterial Repetitive Intergenic Consensus (ERIC‐PCR)

ERIC‐PCR was performed to assess the genetic relatedness among the isolates using the previously described primers: ERIC1‐R (5 ^′^‐ATGTAAGCTCCTGGGGATTCAC‐3 ^′^) and ERIC2‐F (5 ^′^‐AAGTAAGTGACTGGGGTGAGCG‐3 ^′^) [10, 25]. The PCR thermal cycling conditions included an initial denaturation at 95°C for 5 min, followed by 30 cycles of denaturation at 95°C for 60 s, annealing at 59°C for 50 s, and extension at 72°C for 60 s. A final extension step was performed at 72°C for 10 min. The amplified products were separated by electrophoresis on 1.5% agarose gels prepared with 0.5× TBE (Tris‐borate‐EDTA) buffer. After staining with a safe load dye, the DNA bands were visualized under UV light. To ensure the reproducibility of the ERIC‐PCR typing, all DNA extracts were amplified and separated in duplicate. Only clear, reproducible banding profiles were used for cluster analysis. ERIC patterns were analyzed using GelJ software (Version 2.0). Isolates exhibiting a similarity coefficient of 90% or greater were grouped into the same genotype.

### 2.6. Statistical Analysis

Statistical analyses were performed using SPSS software (Version 20; SPSS, Inc.) and GraphPad Prism (Version 8; GraphPad Software, Inc.). Categorical variables were compared using the chi‐square test or Fisher′s exact test, as appropriate, to identify statistically significant associations between variables (*p* < 0.05). Given the exploratory nature of testing multiple resistance phenotypes and multiple resistance/virulence genes against CRISPR‐Cas carriage and biofilm status, we acknowledge an increased risk of false‐negative findings due to limited sample size (*n* = 42). Therefore, *p* values from these univariate comparisons were interpreted cautiously, with emphasis on effect sizes (odds ratios [ORs]) and 95% confidence intervals (CIs). An approximate post hoc sensitivity analysis for 2 × 2 association testing (*α* = 0.05, *n* = 42) suggested limited power to detect moderate effects, supporting cautious interpretation of nonsignificant results, particularly in the context of multiple comparisons. In addition to univariate analyses, a multivariable binary logistic regression model was constructed to identify factors independently associated with vancomycin resistance. Because of the limited sample size and to reduce model overfitting, the number of predictors was restricted a priori. Variables included in the model were biofilm formation (producer vs. nonproducer), presence of CRISPR‐Cas system (positive vs. negative), specimen type (urine vs. nonurine), and presence of the *ermB* gene, selected based on biological plausibility and prior literature. Adjusted ORs with 95% CIs were calculated. Statistical significance was set at *p* < 0.05.

## 3. Results

### 3.1. Bacterial Isolates, Antimicrobial Susceptibility, and Biofilm Formation

In this study, 42 nonduplicate *E. faecalis* isolates were collected from 14 (33.3%) female and 28 (66.7%) male patients, with a mean age of 38.2 ± 7 years (range: 6–58 years). The isolates were obtained from various clinical specimens, including urine (45.2%), blood (26.2%), and wound samples (28.6%). All 42 isolates originated from the following hospital wards: the ICU (45.2%), urology (26.2%), infectious diseases (16.7%), and nephrology (11.9%) (Table 2).

**Table 2:** Antibiotic resistance pattern of the isolates.

	*N* (%)								
	Gentamicin	Vancomycin	Tetracycline	Nitrofurantoin	Ampicillin	Tigecycline	Linezolid	Chloramphenicol	Ciprofloxacin
R	42 (100)	15 (35.7)	20 (47.6)	24 (57.1)	37 (88.1)	17 (40.5)	7 (16.7)	12 (28.6)	29 (69)
I	0	0	8 (19.1)	10 (23.8)	0	0	10 (23.8)	8 (19)	4 (9.5)
S	0	27 (64.3)	14 (33.3)	8 (19.1)	5 (11.9)	25 (59.5)	25 (59.5)	22 (52.4)	9 (21.5)

Abbreviations: I, intermediate; R, resistant; S, susceptible.

Antimicrobial susceptibility testing revealed that linezolid and tigecycline were the most effective antibiotics, each with a susceptibility rate of 59.5% (*n* = 25). In contrast, gentamicin and ampicillin were the least effective, with resistance rates of 100% and 88.1%, respectively. Gentamicin MIC values greater than 500 *μ*g/mL were observed in all isolates (100%), indicating HLGR. The isolates exhibited varying resistance profiles to other antibiotics, as summarized in Table 2. Vancomycin resistance was detected in 35.7% of the isolates, and the related MIC values are presented in Table 3.

**Table 3:** The MICs, biofilm formation, and genetic characteristics of the isolates.

ID	MIC level (*μ*g/mL)		Antibiotic resistance genes							Biofilm formation	Virulence factor genes							CRISPR‐Cas genes				
	VAN	GEN	*ermA*	*ermB*	*ermC*	*vanA*	*Van B*	*aac*(*6* ^′^)*-Ie-aph*(*2* ^″^)*-Ia*	*msrA*		*asaI*	*cylA*	*hyl*	*ace*	*efaA*	*gelE*	*esp*	CR1	CR2	CR3	Cas1	Cas3
1	< 0.25	512	—	+	—	—	—	+	—	Weak	—	—	—	+	+	—	—	+	—	+	—	—
2	4	512	—	+	—	—	—	+	—	Weak	—	—	—	—	+	—	—	+	—	+	—	—
3	> 32	1024	—	+	+	—	—	+	—	Weak	—	—	—	—	+	+	+	+	—	+	—	—
4	4	512	—	+	—	—	—	+	—	Weak	—	—	—	—	+	+	+	+	—	+	—	—
5	> 32	512	—	+	—	+	—	+	—	Weak	—	—	—	+	+	+	—	—	—	+	+	—
6	< 0.25	1024	+	+	—	—	—	+	—	Weak	—	—	—	+	+	+	+	+	—	+	—	—
7	0.5	1024	—	+	—	—	—	+	—	Medium	—	—	—	+	+	+	+	—	—	+	—	—
8	< 0.25	512	—	+	—	—	—	+	—	Weak	—	—	—	+	+	—	+	+	—	+	—	—
9	> 32	512	—	+	—	+	—	+	—	Weak	—	—	—	+	+	+	+	+	—	+	—	—
10	0.25	1024	—	+	—	—	—	+	—	Weak	—	—	—	+	+	+	+	—	—	+	—	—
11	> 32	512	—	+	—	+	—	+	—	Weak	+	—	—	+	+	+	+	—	—	+	+	—
12	0.25	512	—	+	+	—	—	+	—	Weak	+	—	—	+	+	+	—	—	—	+	—	—
13	0.25	512	+	+	—	—	—	+	—	Weak	—	—	—	+	+	—	+	—	—	+	+	—
14	< 0.25	512	+	+	—	—	—	+	—	None	+	—	—	+	+	—	+	+	—	+	—	—
15	2	512	—	+	—	—	—	+	—	Weak	+	—	—	+	+	—	—	—	—	+	+	—
16	32	512	—	+	—	—	—	+	—	Weak	+	—	—	+	+	—	+	+	—	+	—	—
17	> 32	1024	+	+	—	+	—	+	—	Weak	+	—	—	+	+	—	+	—	—	+	+	—
18	> 32	512	—	+	—	+	—	+	—	Weak	+	—	—	—	+	+	+	+	—	+	—	—
19	4	512	+	+	—	—	—	+	—	Weak	—	—	—	—	+	+	+	+	—	+	—	—
20	0.5	1024	—	—	—	—	—	+	—	Weak	+	—	—	—	+	—	+	—	—	+	+	—
21	32	512	—	—	+	—	—	+	—	Weak	+	—	—	—	+	+	+	—	—	+	+	—
22	0.25	512	—	—	—	—	—	+	—	Weak	+	—	—	+	+	—	+	+	—	+	+	—
23	4	512	—	+	—	—	—	+	—	Weak	+	—	—	+	+	+	+	—	—	+	+	—
24	0.25	1024	—	+	—	—	—	+	—	Weak	+	—	—	+	+	—	—	+	—	+	—	—
25	> 32	512	—	—	—	+	—	+	—	Weak	—	—	—	—	+	+	—	+	—	—	—	—
26	< 0.25	512	—	+	—	—	—	+	—	Weak	—	—	—	+	+	+	+	+	—	+	—	—
27	< 0.25	512	—	—	—	—	—	+	—	Weak	—	—	—	—	+	+	+	—	—	—	—	—
28	< 0.25	512	—	+	—	—	—	+	—	Medium	+	—	—	—	+	+	—	—	—	—	—	—
29	32	512	—	+	—	—	—	+	—	Weak	+	—	—	—	+	—	+	—	—	—	—	—
30	4	512	—	+	—	—	—	+	—	Weak	+	—	—	+	+	+	+	+	—	+	—	—
31	< 025	512	+	—	—	—	—	+	—	Weak	—	—	—	—	+	+	+	—	—	+	—	—
32	> 32	1024	+	+	+	—	—	+	—	Weak	+	—	—	+	+	+	+	+	—	+	—	—
33	32	512	—	+	—	—	—	+	—	Weak	+	—	—	+	+	+	+	+	—	—	—	—
34	2	512	+	—	—	—	—	+	—	None	+	—	—	+	+	+	+	+	—	+	—	—
35	> 32	512	+	—	—	+	—	+	—	Weak	+	—	—	+	+	+	+	—	—	+	+	—
36	< 0.25	512	+	—	—	—	—	+	—	Weak	+	—	—	+	+	+	+	+	—	+	—	—
37	< 0.25	512	+	+	+	—	—	+	—	Weak	+	—	—	+	+	+	+	—	+	+	—	—
38	< 0.25	512	+	+	+	—	—	+	—	Weak	+	—	—	+	+	+	—	—	+	—	—	—
39	0.25	512	+	+	+	—	—	+	—	None	+	—	—	+	+	+	—	—	+	+	—	—
40	32	512	+	+	+	—	—	+	—	Weak	+	—	—	+	+	+	—	+	+	+	—	—
41	4	1024	+	+	+	—	—	+	—	Weak	—	—	—	—	—	—	—	—	—	—	—	—
42	> 32	512	+	+	+	+	—	+	—	Weak	—	—	—	—	—	—	—	—	—	—	—	—

Abbreviations: Cas1, CRISPR1‐cas csn1; Cas3, CRISPR3‐cas csn1; CR1,: CRISPR1‐cas loci;, CR2,: CRISPR2 loci; CR3, CRISPR3‐cas loci; GEN, gentamicin; VAN, vancomycin.

Overall, 39 isolates (92.8%) were biofilm producers, of which 37 (88.1%) and 2 (4.7%) produced weak and moderate biofilms, respectively. No biofilm production was observed in three isolates (7.1%). No significant association was found between biofilm production intensity and antimicrobial susceptibility (Table 4).

**Table 4:** Relationship between the biofilm intensity and antibiotic susceptibility.

Biofilm (*N*)	*N* (%)																										
	Gentamicin			Vancomycin			Tetracycline			Nitrofurantoin			Ampicillin			Linezolid			Chloramphenicol			Ciprofloxacin			Tigecycline		
	S	I	R	S	I	R	S	I	R	S	I	R	S	I	R	S	I	R	S	I	R	S	I	R	S	I	R
Medium (2)	0	0	2 (100)	2 (100)	0	0	1 (50)	1 (50)	0	0	0	2 (100)	0	0	2 (100)	1 (50)	0	1 (50)	0	0	2 (100)	2 (100)	0	0	2 (100)	0	0
Weak (37)	0	0	37 (100)	22 (59.5)	0	15 (40.5)	13 (35)	7 (19)	17 (46)	8 (21)	8 (21)	21 (58)	5 (13)	0	32 (87)	22 (60)	10 (27)	5 (13)	20 (54)	7 (19)	10 (27)	6 (16)	4 (12)	27 (72)	21 (56)	0	16 (44)
None (3)	0	0	3 (100)	3 (100)	0	0	0	0	3 (100)	0	2 (66)	1 (33)	0	0	3 (100)	2 (66)	0	1 (33)	2 (66)	1 (33)	0	1 (33)	0	2 (66)	2 (66)	0	1 (33)
*p* value	—			0.253			0.198			0.999			0.999			0.360			0.210			0.094			0.253		

### 3.2. Distribution of the Investigated Genes

The *efaA* gene was the most prevalent virulence gene, identified in 40 isolates (95.2%), followed by *ace* (66.6%), *esp* (69%), *gelE* (66.6%), and *asa1* (57.1%). The *cylA* and *hyl* genes were not detected in any of the isolates. The most common virulence gene combination was the simultaneous carriage of *efaA*, *ace*, and *esp*.

Regarding antimicrobial resistance genes, the *aac*(*6*  ^′^)*-Ie-aph*(*2*  ^″^)*-Ia* gene was detected in all isolates (100%), followed by *ermB* (78.6%), *ermA* (38.1%), and *ermC* (23.8%). The *vanA* gene was found in eight isolates (19%), whereas *vanB* and *msrA* were not detected. The presence of the *vanA* gene in vancomycin‐resistant isolates was statistically significant (*p* : 0.001) and all *vanA*‐positive isolates showed resistance to vancomycin (Table 3).

**Figure 1:**
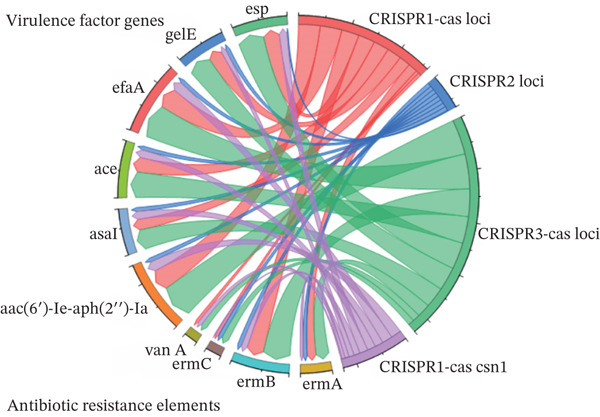
Association between the occurrence of CRISPR‐Cas positive and antibiotic resistance and virulence genes.

Among CRISPR‐Cas loci, CRISPR3 was the most frequently detected (80.95%), followed by CRISPR1 (50%), CRISPR1‐cas csn1 (23.8%), and CRISPR2 (9.5%). Thirty‐seven isolates (88.1%) harbored at least one CRISPR‐related gene, whereas the CRISPR3‐cas csn1 gene was not detected in any isolate. Five isolates (11.9%) lacked all CRISPR‐Cas genes. The antimicrobial resistance patterns and CRISPR‐Cas genes are presented in Table 5. No significant differences were observed between the presence or absence of CRISPR‐Cas genes and antibiotic resistance or virulence factors, except for *ace*, *efaA*, and *esp* genes. The presence of these virulence factors in CRISPR3‐positive isolates was statistically significant (*p* < 0.05) (Tables 6, 7, and 8 and Figure 1). In multivariate logistic regression analysis, none of the investigated variables, including biofilm formation, CRISPR‐Cas presence, specimen type, and *ermB* gene, showed a statistically significant independent association with vancomycin resistance (*p* > 0.05).

**Table 5:** Antibiotic resistance pattern and CRISPR‐Cas genes.

ID	Resistance pattern									CRISPR‐Cas genes				
	GEN	VAN	TET	NIT	AMP	LNZ	CHO	TGC	CIP	CRISPR1‐cas loci	CRISPR2 loci	CRISPR3‐cas loci	CRISPR1‐cas csn1	CRISPR3‐cas csn1
1	R	S	R	R	R	S	R	S	R	+	—	+	—	—
2	R	S	S	R	R	I	S	R	R	+	—	+	—	—
3	R	R	R	R	R	S	R	S	S	+	—	+	—	—
4	R	S	R	I	R	R	S	S	R	+	—	+	—	—
5	R	R	S	R	R	S	S	R	R	—	—	+	+	—
6	R	S	R	I	R	I	S	S	R	+	—	+	—	—
7	R	S	I	R	R	S	R	S	S	—	—	+	—	—
8	R	S	R	S	S	S	S	R	R	+	—	+	—	—
9	R	R	S	I	R	S	I	S	R	+	—	+	—	—
10	R	S	R	R	R	I	S	S	R	—	—	+	—	—
11	R	R	R	R	R	S	I	R	R	—	—	+	+	—
12	R	S	S	S	R	S	R	R	R	—	—	+	—	—
13	R	S	I	I	R	R	S	R	I	—	—	+	+	—
14	R	S	R	R	R	S	S	S	R	+	—	+	—	—
15	R	S	S	R	R	I	I	S	S	—	—	+	+	—
16	R	R	I	I	S	S	S	R	R	+	—	+	—	—
17	R	R	S	R	R	S	R	S	S	—	—	+	+	—
18	R	R	R	R	R	S	S	S	R	+	—	+	—	—
19	R	S	S	R	R	S	I	R	R	+	—	+	—	—
20	R	S	R	S	R	R	S	R	I	—	—	+	+	—
21	R	R	R	R	R	I	R	S	R	—	—	+	+	—
22	R	S	R	R	R	S	S	R	R	+	—	+	+	—
23	R	S	I	S	R	R	R	S	R	—	—	+	+	—
24	R	S	R	I	R	S	R	S	I	+	—	+	—	—
25	R	R	S	R	R	I	S	R	R	+	—	—	—	—
26	R	S	R	R	S	S	S	S	R	+	—	+	—	—
27	R	S	I	I	R	S	I	S	R	—	—	—	—	—
28	R	S	S	R	R	R	R	S	S	—	—	—	—	—
29	R	R	R	R	R	S	R	R	R	—	—	—	—	—
30	R	S	S	S	S	S	I	S	R	+	—	+	—	—
31	R	S	R	R	R	I	S	R	R	—	—	+	—	—
32	R	R	I	I	R	S	S	S	R	+	—	+	—	—
33	R	R	S	R	R	I	I	R	S	+	—	—	—	—
34	R	S	I	R	R	S	R	S	R	+	—	+	—	—
35	R	R	R	I	R	R	S	S	S	—	—	+	+	—
36	R	S	R	S	S	I	S	R	R	+	—	+	—	—
37	R	S	S	R	R	S	S	S	I	—	+	+	—	—
38	R	S	I	R	R	R	R	S	S	—	+	—	—	—
39	R	S	R	R	R	S	I	R	R	—	+	+	—	—
40	R	R	S	S	R	S	S	S	S	+	+	+	—	—
41	R	S	S	I	R	I	S	S	R	—	—	—	—	—
42	R	R	R	S	R	S	S	R	R	—	—	—	—	—

**Table 6:** Association between the occurrence of CRISPR‐Cas positive/negative and antibiotic resistance.

Antibacterial agent	Resistance rate *N* (%)		*p* value
	CRISPR‐Cas positive (37)	CRISPR‐Cas negative (5)	
Gentamicin	37 (100)	5 (100)	—
Vancomycin	13 (35.1)	2 (40)	0.831
Tetracycline	18 (48.6)	2 (40)	0.999
Nitrofurantoin	22 (59.5)	2 (40)	0.636
Ampicillin	32 (86.5)	5 (100)	0.612
Linezolid	6 (16.2)	1 (20)	0.831
Chloramphenicol	10 (27)	2 (40)	0.547
Tigecycline	15 (40.5)	2 (40)	0.982
Ciprofloxacin	25 (67.6)	4 (80)	0.572

**Table 7:** Association between the occurrence of CRISPR‐Cas and antibiotic resistance genes.

CRISPR/Cas *N* (%)	Presence of antibiotic resistant genes *N* (%)				
	*ermA* 16 (38.1)	*ermB* 33 (78.6)	*ermC* 10 (23.8)	*vanA* 8 (19)	*aac*(*6* ^′^)*-Ie-aph*(*2* ^″^)*-Ia* 42 (100)
CRISPR/Cas positive 37 (88.1)	14 (87.5)	29 (87.87)	8 (80)	7 (87.5)	37 (88.09)
CRISPR/Cas negative 5 (11.9)	2 (12.5)	4 (12.13)	2 (20)	1 (12.5)	5 (11.91)
*p* value	0.641	0.712	0.341	0.673	—

*Note: vanB* and *msrA*: not detected in any isolate.

**Table 8:** Association between the occurrence of CRISPR‐Cas positive and negative and virulence factor genes.

Virulence factor		CRISPR/Cas no. (%)								
		CRISPR1‐Cas loci		*p* value	CRISPR3‐Cas loci		*p* value	CRISPR1‐Cas csn1		*p* value
		+	−		+	−		+	−	
*asaI*	+	11	13	0.53	20	4	0.65	8	16	0.09
		(27)	(30)		(47)	(10)		(19)	(38.5)	
	−	10	8		14	4		2	16	
		(24)	(19)		(33)	(10)		(4)	(38.5)	
*ace*	+	15	13	0.51	26	2	0.005	8	20	0.3
		(35)	(30)		(62)	(4)		(19)	(48)	
	−	6	8		8	6		2	12	
		(16)	(19)		(19)	(15)		(4)	(29)	
*efaA*	+	21	19	0.14	34	6	0.002	10	30	0.41
		(50)	(45)		(81)	(15)		(24)	(71)	
	−	0	2		0	2		0	2	
			(5)			(4)			(4)	
*gelE*	+	14	14	> 0.99	23	5	> 0.78	5	23	0.2
		(33)	(33)		(55)	(12)		(12)	(55)	
	−	7	7		11	3		5	9	
		(17)	(17)		(26)	(7)		(12)	(21)	
*esp*	+	16	13	0.31	26	3	0.03	8	21	0.39
		(38)	(30)		(62)	(7)		(19)	(50)	
	−	5	8		8	5		2	11	
		(12)	(20)		(19)	(12)		(4)	(27)	

### 3.3. ERIC‐PCR Typing

**Figure 2:**
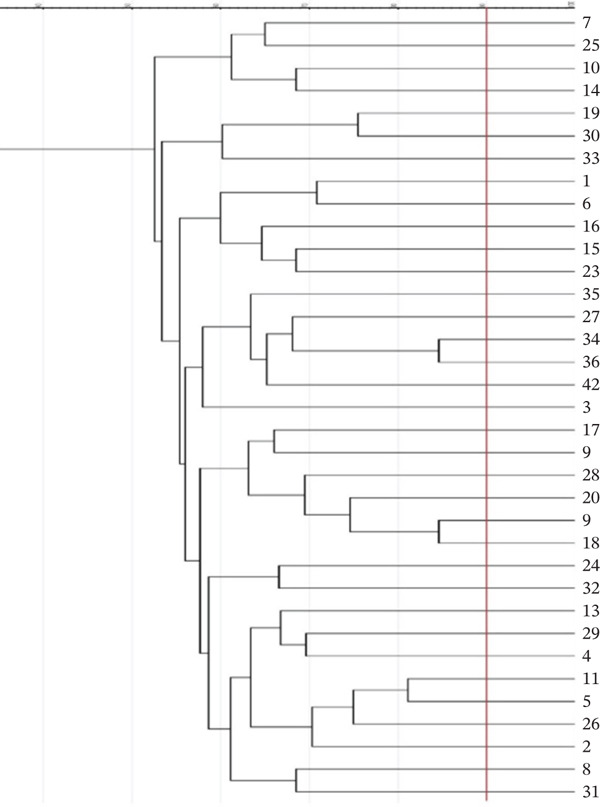
Dendrogram generated from ERIC‐PCR fingerprinting of the isolates.

As shown in Figure 2, a high degree of genetic diversity was observed among the isolates. Based on a similarity coefficient of ≥ 90%, 35 isolates were classified into 35 distinct ERIC types, with no two isolates sharing the same genotype. Additionally, seven isolates failed to produce banding patterns following ERIC‐PCR and were therefore classified as nontypeable. The four isolates positive for the CRISPR2 locus were also included in the nontypeable group.

## 4. Discussion

*E. faecalis* is an opportunistic pathogen capable of causing severe infections, particularly within healthcare settings. Its pathogenicity is characterized by both intrinsic and acquired antimicrobial resistance, alongside various virulence factors that facilitate adherence and invasiveness [5]. In the current study, 42 *E. faecalis* isolates from various clinical samples were evaluated for virulence factors, biofilm production, and antibiotic resistance. The phenotypic analysis of antibiotic susceptibility tests revealed a high resistance rate of 100% to gentamicin and 88.1% to ampicillin. In contrast, linezolid and chloramphenicol demonstrated the highest effectiveness, with resistance rates of 16.7% and 28.6%, respectively. Moreover, the results of broth microdilution method showed the high resistance to gentamicin and vancomycin with MIC levels greater than 500 and 32 *μ*g/mL, respectively. Antibiotic stewardship and patterns of antibiotic resistance in *E. faecalis* strains vary across different geographical regions based on clinical samples, leading to a range of findings in studies on this topic. Previous studies performed have reported high resistance rates to gentamicin and ampicillin among *E. faecalis* isolates primarily obtained from urine samples [5, 26, 27]. Meanwhile, two studies conducted in China and Iran on urine samples reported high resistance rates of *E. faecalis* strains to tetracycline, minocycline, cotrimoxazole, and vancomycin [28, 29]. A recent meta‐analysis conducted by Guan et al. [30] found significant differences in antibiotic resistance rates for chloramphenicol, ampicillin, gentamicin, erythromycin, tetracycline, and vancomycin across various countries. This research highlights a growing prevalence of drug‐resistant *E. faecalis* strains, especially those resistant to chloramphenicol, tetracycline, and linezolid. Furthermore, their analysis suggests that daptomycin and tigecycline could be effective therapeutic options for treating clinical infections caused by *E. faecalis*.

The genomic analysis of antibiotic resistance genes in the current study showed a high prevalence of *ermB* (78.6%) and *aac*(*6*  ^′^)*-Ie-aph*(*2*  ^″^)*-Ia* (100%) genes among the *E. faecalis* isolates. Our findings are similar to several other investigations reporting a high prevalence of *aac*(*6*  ^′^)*-Ie-aph*(*2*  ^″^)*-Ia* and *ermB* genes among VRE isolates [10, 29, 31, 32]. The 100% prevalence of the *aac*(*6*  ^′^)*-Ie-aph*(*2*  ^″^)*-Ia* gene paired with universal phenotypic HLGR offers critical regional surveillance insights and underscores a severe clinical warning. These findings confirm the absolute loss of traditional synergistic therapeutic regimens combining cell‐wall active agents (e.g., ampicillin or glycopeptides) with aminoglycosides in our clinical center. Consequently, alternative therapeutic pathways, such as linezolid and tigecycline, which demonstrated the highest clinical efficacy in our susceptibility testing (59.5%) should be prioritized for managing severe enterococcal infections locally. In recent years, the extensive use of macrolides has resulted in the emergence of resistant *Enterococcus* strains to these two important classes of antibiotics. Although Zalipour et al. [31] reported a 30.2% prevalence of the *msrA* gene, our findings are consistent with earlier studies that did not identify the *msrA* gene in any of the isolates examined [10, 32]. MsrA/MsrB efflux pumps are related to the ABC efflux pump families and are responsible for conferring significant resistance to macrolides [33]. In the present study conducted, 35.7% of strains were VRE. However, genotypic analysis showed that a lower rate (19%) of the isolates possessed the *vanA* gene that is similar to other studies from Iran [5, 31, 32]. Other studies have shown a lower prevalence of *vanA/B* genes in *E. faecalis* isolates from blood and burn wound samples [10, 34]. The comprehensive meta‐analysis showed that *E. faecalis* strains had a 4.3% proportion of vancomycin resistance with significant heterogeneity among different reports [30]. These studies have primarily focused on the phenotypic resistance to vancomycin, but a comprehensive understanding of the genes associated with resistance to this antibiotic is still lacking in the literature.

In our study, 92.8% of the isolates exhibited weak to moderate biofilm formation, similar to the findings of Khdir et al. [35]. Although several studies have reported high levels of biofilm production among *E. faecalis* strains, particularly observed in bacteria isolated from urine samples [5, 26, 28, 36]. In our study, no significant correlation was found between the biofilm intensity and antibiotic susceptibility. Factors such as poor penetration of antibiotics, adaptive responses to stress, nutrient limitation, and persister cells within biofilms all contribute to the emergence of antibiotic resistance [37].

In the current study, the pattern of virulence factor genes indicated a high frequency of *efaA* (95.2%), *asa1* (57.1%), *esp* (69%), and *gelE*, *ace* (66.6%) in *E. faecalis* isolates. Molecular analysis by Koleini et al. [5] showed that nearly 80% of *E. faecalis* isolates carried the *asa1* gene. Biofilm formation is a multifactorial process, and its development may be correlated with the presence of various virulence genes that contribute to biofilm stability and aggregation by hydrolyzing the extracellular components. The main adhesion factors of *E. faecalis* include Asa, Ace, EfaA, and Esp [38]. Aggregation substance (*asa1* encoded) is a virulence factor that promotes bacterial adhesion to host tissues, particularly renal tubular cells, and results in the development of *E. faecalis* biofilms in vitro. The collagen adhesin Ace is vital in laboratory‐induced urinary tract infections caused by *E. faecalis* [39]. Mutant strains of this bacterium that lack this adhesin are less virulent than wild‐type strains [40]. The Esp of *E. faecalis* is known for facilitating surface attachment, which in turn contributes to biofilm development. It has been shown that mutants deficient in Esp insertion–deletion create unstructured and weak biofilms in contrast to the strong biofilms observed in the wild type [41].

Two studies from China reported a high frequency of the *gelE* and *efa* genes among *E. faecalis* isolates [29, 34]. Enterococcal GelE enzyme degrades gelatin, causing tissue damage and facilitating the spread of these bacteria within their host. Moreover, GelE plays a substantial role in biofilm development by gathering cells to form microcolonies and initiating the early stages of this process [36]. Although no significant correlation was found between adhesion factors and the severity of biofilm formation in our study, the results of several studies investigating biofilm formation by *E. faecalis* isolates derived from urine samples suggest a significant correlation between strong biofilm formation and the presence of *efa*, *gelE*, *ace*, and *esp* genes [5, 26–28, 36, 42]. Although our multivariate models did not show statistically significant associations overall, clear epidemiological patterns appeared once we analyzed the data by clinical setting. Specifically, isolates from intensive care and surgical units showed a greater concentration of MDR profiles and virulence gene combinations, such as *efaA*, *ace*, and *esp* than those found in general medicine wards. This suggests that the strong selective pressure in critical‐care settings may help preserve certain adaptive traits. By keeping both biofilm‐forming ability and genetic resistance, *E. faecalis* may survive better in these hospital environments.

The investigated isolates exhibited a complete absence of the secreted exotoxin genes *cylA* and *hyl* (0.0%). This lack of toxin‐encoding genes suggests that the pathogenesis of local *E. faecalis* strains is driven primarily by structural cell‐wall adhesins (e.g., *efaA* and *ace*) and the formation of protective biofilms, which facilitate colonization and persistence in hospital environments, rather than through the secretion of matrix‐degrading toxins.

In the current study, CRISPR3 was the gene most frequently detected at 80.95%, whereas CRISPR1 was present in 50% of the isolates. Moreover, 61.9% and 4.76% of *E. faecalis* isolates carried two and three CRISPR genes, respectively. Considering the global challenge of antimicrobial resistance, creating innovative approaches for managing infections and preventing the dissemination of antimicrobial resistance is crucial. There is an urgent need for *E. faecalis* strains that possess a functional CRISPR‐Cas system. These strains can inhibit the uptake of mobile genetic elements, particularly plasmids, limiting the spread of antibiotic resistance genes [17]. Due to its ability to target and cleave DNA sequences associated with antibiotic resistance, the CRISPR/Cas system has been developed into a novel gene‐editing tool preventing and managing bacterial drug resistance [43]. Previously, the genetic structure analysis of 110 *Enterococcus* strains demonstrated that 39 strains (35.45%) had a complete CRISPR‐Cas system, with 59.68% being type 2‐A and 33.87% being Type 2‐C [44]. The findings presented by Huo et al. [45] indicated that the selection of antibiotics leads to a diminished function of the CRISPR‐Cas system in *E. faecalis*, which in turn promotes the uptake of further resistance plasmids by this bacterium. Similarly, Tao et al. [43] found orphan CRISPR2 to be the most prevalent CRISPR‐Cas system among *Enterococcus* isolates (42.0%). The findings showed that CRISPR‐Cas negative isolates exhibited significantly higher resistance to erythromycin, ampicillin, levofloxacin, vancomycin, tetracycline, streptomycin, gentamicin, and rifampicin compared with CRISPR‐Cas positive isolates.

In the current study, 40% (two out of five) of CRISPR‐Cas negative isolates were resistant to vancomycin, tetracycline, nitrofurantoin, chloramphenicol, and tigecycline, and no statistically significant difference was found between the CRISPR‐Cas–positive and –negative *E. faecalis* isolates regarding antibiotic resistance. In the study by Gholizadeh et al. [14], CRISPR loci were frequently found in *E. faecalis* strains susceptible to teicoplanin, gentamicin, erythromycin, and tetracycline, which were isolated from urinary tract infections and dental root canals. They observed fewer CRISPR loci in strains positive for *vanA*, *tetM*, *ermB*, *aac*(*6*  ^′^)*-Ie-aph*(*2*  ^″^)*-Ia*, suggesting a negative relationship between CRISPR‐Cas loci and antibiotic resistance.

In our study, the high rates of multidrug resistance and virulence factors found in the isolates highlight how effectively they adapt as resilient opportunistic pathogens in hospitals. Interestingly, although the CRISPR‐Cas system is known to act as an immune barrier against mobile genetic elements carrying resistance genes, we found no statistically significant link between CRISPR‐Cas genes and phenotypic or genotypic resistance. Earlier research often points to a strict inverse relationship where MDR enterococci lack working CRISPR‐Cas systems due to intense antibiotic pressure [46, 47]. However, recent large‐scale genomic studies suggest a more nuanced picture [48]. The lack of association observed in our study regarding resistance may be explained by the presence of nonfunctional or compromised CRISPR‐Cas machinery; even when CRISPR‐Cas loci like CRISPR3 (80.95%) or CRISPR1 (50%) are frequently detected, they may lack the full complement of functional *cas* genes required for interference, rendering them unable to prevent the horizontal gene transfer of resistance determinants like *aac*(*6*  ^′^)*-Ie-aph*(*2*  ^″^)*-Ia* or *vanA* [48, 49]. Furthermore, under heavy antibiotic pressure, enterococci can temporarily tolerate plasmids targeted by CRISPR or develop mutations that allow them to bypass CRISPR‐mediated immunity [50].

Crucially, our statistical analysis revealed a significant positive correlation (*p* < 0.05) between the presence of the CRISPR3 locus and specific virulence genes (*ace*, *efaA*, and *esp*), which contrasts with the lack of association found among other virulence traits. In a previous research, an inverse significant correlation was detected between *esp*, *gelE*, and CRISPR‐*cas* loci, whereas direct correlations were found in the case of *gelE*, *hyl*, *cylA* (among CRISPR‐loci 1 and 3), *ace*, and *asa1* [46]. One of the earliest studies examining the relationship between CRISPR‐Cas systems and the presence of virulence factors assessed 62 *Enterococcus* strains isolated from clinical, food, and environmental sources. The study reported that the occurrence of CRISPR elements correlated with a reduced number of virulence factors. Specifically, the presence of the cytolysin operon, as well as the genes encoding pheromone and aggregation substance, was significantly associated with the absence of cas [51]. In a recent study, the virulence gene *esp* was found to be less common in CRISPR‐positive isolates than in CRISPR‐negative ones. Furthermore, the association between the presence of CRISPR‐Cas and the absence of the *gelE* gene was significant [43]. Our observation of a high CRISPR3 prevalence (80.95%), alongside a lack of an inverse association with multidrug resistance and notable prevalence of virulence traits, was in contrast to the patterns previously established by Palmer and Gilmore [16] and Tao et al. [43]. This fascinating biological interplay suggests that the relationship between CRISPR immunity and pathogenicity may be more complicated. Instead, the CRISPR3 locus may actively coexist with, or fail to target, these specific structural and adherence factors. This may occur because these genes are carried on highly conserved chromosomal regions or stable pathogenicity islands rather than on the unstable, transient plasmids that CRISPR spacers usually target [48, 49]. Furthermore, because of the high ability of biofilm production (92.8%) in the studied isolates, the biofilm matrix itself might act as a physical shield for these bacterial communities, driving localized horizontal gene transfer of these virulence factors while allowing CRISPR‐carrying strains to thrive under selective clinical conditions [49]. This highlights the incredibly adaptive evolutionary strategies of *Enterococcus* species. The presence of a specific CRISPR locus like CRISPR3 does not mean foreign pathogenic material is automatically blocked. Instead, it may offer a clear survival advantage under intense selective pressure.

The ability of these bacteria to both form extensive biofilms (92.8%) and maintain complex drug resistance points to a highly coordinated survival strategy. In higher order organisms (eukaryotes), the mTOR (mechanistic target of rapamycin) pathway acts as a central control hub that senses nutrient levels and manages stress to keep the cell alive [52–54]. Our studied *Enterococcus* isolates seem to use a very similar nutrient‐sensing logic. When faced with harsh hospital conditions and antibiotic stress, they must carefully ration their energy. They do this using bacterial communication networks, like the (p)ppGpp stringent response and two‐component regulatory systems. These networks act just like the mTOR framework: They detect environmental dangers and order the cell to temporarily pause everyday growth. Instead, the cell redirects its energy into heavy‐duty survival tactics, such as switching on major resistance genes (*vanA* and *aac*(*6*  ^′^)*-Ie-aph*(*2*  ^″^)*-Ia*) and building protective biofilms. This cross‐kingdom comparison reveals that these bacteria do not just acquire resistance traits at random; it is a plan to balance growth and defense under stress.

In current study, the results of ERIC‐PCR analysis demonstrated high heterogeneity among the isolates as they were classified into 35 different ERIC types. None of the isolates were included in the same genotype, which is probably due to the difference in clinical samples from different wards and the time period of the sampling during 1 year. As we discussed before, despite most of the isolates exhibiting weak to moderate biofilm formation and CRISPR 3, their antibiotic resistance and virulence gene patterns varied significantly, suggesting the heterogeneity of the isolates. In the study by Zalipour et al. [31], 14 ERIC‐types were found among 53 vancomycin‐resistant *E. faecalis* isolates collected from urine. The authors suggest that the high degree of genetic similarity observed among the majority of the analyzed isolates is likely the result of sample collection from the same hospital ICU or internal ward. In a previous study, genotyping of 50 *E. faecalis* isolates isolated from stool samples showed 25 ERIC types. The *E. faecalis* isolates were mainly categorized into 10 clusters, comprising two major and eight minor clusters. No significant differences were observed between the two major clusters regarding antimicrobial resistance and resistance genes [55]. Another study from Iran revealed high diversity (34 ERIC types) among 57 *Enterococcus* isolates collected from burn wounds [10]. ERIC‐PCR analysis of 25 *E. faecalis* isolates identified two major clusters, with Cluster B accounting for 72% of the isolates. However, no correlation was found between the virulence profiles and the phylogenetic groups of the isolates [56]. These discrepancies in findings are probably due to the significant variability of *E. faecalis*, which could be associated with differences in its nucleotide sequences. The notable genetic diversity among the isolates could be crucial for the survival of different *E. faecalis* strains in the studied setting.

We found that seven *E. faecalis* isolates (16.7%) could not be typed by ERIC‐PCR because no amplicon bands were produced. Since these same DNA samples performed well in all other virulence and resistance gene assays, we can rule out poor DNA quality as a factor. Instead, this suggests significant genomic variation within the binding sites targeted by the ERIC primers. This “failure” to amplify actually serves as evidence of the underlying genomic diversity among these regional clinical strains. Intriguingly, all isolates harboring the CRISPR2 locus were found to be nontypeable via ERIC‐PCR. This absolute lack of amplification suggests a distinct genomic divergence within this specific sublineage, potentially due to mutations or deletions at the highly conserved repetitive intergenic consensus primer‐binding sites. This structural variation underscores the genomic heterogeneity of local *E. faecalis* strains and supports the argument for high‐resolution sequencing methods to map these lineages fully.

## 5. Conclusion

This study provides a comprehensive analysis of clinical *E. faecalis* isolates, revealing significant clinical challenges. The high prevalence of gentamicin and vancomycin resistance, compounded by notable biofilm production, underscores the difficulty in managing these infections. Furthermore, the coexistence of multiple virulence traits and CRISPR‐Cas systems highlights the complex genetic landscape of *E. faecalis*. The substantial genetic diversity observed among these isolates necessitates the development of more targeted and efficient therapeutic strategies to combat enterococcal infections effectively.

### 5.1. Study Limitations

Although this study provides essential epidemiological insights into the distribution of CRISPR‐Cas systems, virulence factors, and resistance genes in *Enterococcus* isolates, several limitations warrant acknowledgment. Methodologically, our reliance on PCR‐based detection allows for the identification of CRISPR‐Cas loci but does not confirm their functional transcriptional activity or sequencing integrity. Consequently, we cannot distinguish between active adaptive immune networks and degraded, nonfunctional orphan arrays; we have therefore refined our terminology to “CRISPR loci carriage” to reflect this distinction. Furthermore, our characterization of mobile genetic elements was limited to some specific resistance genes without complete sequencing of the flanking plasmid or transposon backbones, which constrains our ability to trace precise horizontal gene transfer events. Similarly, although ERIC‐PCR offered a cost‐effective screening tool, its lower discriminatory power compared with high‐resolution methods—such as MLST or WGS—means that true clonal complexes and high‐resolution phylogenetic lineages could not be fully resolved.

Additionally, the study′s generalizability is constrained by its localized, cross‐sectional design involving a relatively small cohort (*n* = 42) from two regional hospitals. This limited sample size, alongside potential genotypic heterogeneity from mixed clinical specimens, may reduce the statistical power to detect low‐frequency associations between resistance phenotypes and CRISPR carriage. Future multicenter studies utilizing high‐resolution genotyping (e.g., MLST or WGS) and functional assays are necessary to confirm the evolutionary dynamics and mechanistic drivers of resistance within these enterococcal populations.

## Author Contributions

H.K. contributed to the study conception, design, and supervision. Data collection and analysis were performed by M.N. and S.G. The manuscript was written by S.K., H.H., and M.N. M.N. and H.H. have contributed to the work equally and should be regarded as co‐first authors.

## Funding

No funding was received for this manuscript.

## Disclosure

All authors approved the final manuscript.

## Conflicts of Interest

The authors declare no conflicts of interest.

## Data Availability

Data are available within the manuscript or upon request from the corresponding author.

## References

[1] Georges M. , Odoyo E. , Matano D. , Tiria F. , Kyany′a C. , Mbwika D. , Mutai W. C. , and Musila L. , Determination of *Enterococcus faecalis* and *Enterococcus faecium* Antimicrobial Resistance and Virulence Factors and Their Association With Clinical and Demographic Factors in Kenya, Journal of Pathogens. (2022) 2022, 3129439, 10.1155/2022/3129439. 36405031 PMC9668473

[2] Noskin G. A. , Peterson L. R. , and Warren J. R. , *Enterococcus faecium* and *Enterococcus faecalis* Bacteremia: Acquisition and Outcome, Clinical Infectious Diseases. (1995) 20, no. 2, 296–301, 10.1093/clinids/20.2.296, 7742433. 7742433

[3] Zacharopoulos G. V. , Manios G. A. , Papadakis M. , Koumaki D. , Maraki S. , Kassotakis D. , Bree E. , and Manios A. , Comparative Activities of Ampicillin and Teicoplanin Against *Enterococcus faecalis* Isolates, BMC Microbiology. (2023) 23, no. 1, 10.1186/s12866-022-02753-1, 36609223. PMC9817409 36609223

[4] Beganovic M. , Luther M. K. , Rice L. B. , Arias C. A. , Rybak M. J. , and LaPlante K. L. , A Review of Combination Antimicrobial Therapy for *Enterococcus faecalis* Bloodstream Infections and Infective Endocarditis, Clinical Infectious Diseases. (2018) 67, no. 2, 303–309, 10.1093/cid/ciy064, 29390132. 29390132 PMC6248357

[5] Koleini M. , Mosadegh A. , Madadizadeh F. , and Heidari H. , Assessment of Factors Contributing to Infection Severity and High Levels of Drug Resistance in Clinical Enterococcus Isolates, Journal of Clinical Laboratory Analysis. (2025) 39, no. 14, e70063, 10.1002/jcla.70063, 40432202. 40432202 PMC12287666

[6] Shahini Shams Abadi M. , Taji A. , Salehi F. , Kazemian H. , and Heidari H. , High-Level Gentamicin Resistance Among Clinical Isolates of Enterococci in Iran: A Systematic Review and Meta-Analysis, Folia Medica. (2021) 63, no. 1, 15–23, 10.3897/folmed.63.e53506, 33650391. 33650391

[7] Mareković I. , Markanović M. , Lešin J. , and Ćorić M. , Vancomycin-Resistant Enterococci: Current Understandings of Resistance in Relation to Transmission and Preventive Strategies, Pathogens.(2024) 13, no. 11, 10.3390/pathogens13110966, 39599519. PMC11597547 39599519

[8] Aung M. S. , Urushibara N. , Kawaguchiya M. , Ohashi N. , Hirose M. , Kudo K. , Tsukamoto N. , Ito M. , and Kobayashi N. , Antimicrobial Resistance, Virulence Factors, and Genotypes of *Enterococcus faecalis* and *Enterococcus faecium* Clinical Isolates in Northern Japan: Identification of optrA in ST480 E. faecalis, Antibiotics. (2023) 12, no. 1, 10.3390/antibiotics12010108. PMC9855154 36671309

[9] Sharifzadeh Peyvasti V. , Mohabati Mobarez A. , Shahcheraghi F. , Khoramabadi N. , Razaz Rahmati N. , and Hosseini D. R. , High-Level Aminoglycoside Resistance and Distribution of Aminoglycoside Resistance Genes Among Enterococcus spp. Clinical Isolates in Tehran, Iran, Journal of Global Antimicrobial Resistance. (2020) 20, 318–323, 10.1016/j.jgar.2019.08.008. 31542554

[10] Heidari H. , Emaneini M. , Dabiri H. , and Jabalameli F. , Virulence Factors, Antimicrobial Resistance Pattern and Molecular Analysis of Enterococcal Strains Isolated From Burn Patients, Microbial Pathogenesis. (2016) 90, 93–97, 10.1016/j.micpath.2015.11.017, 26620079. 26620079

[11] Fiore E. , Van Tyne D. , and Gilmore M. S. , Pathogenicity of Enterococci, Microbiology Spectrum. (2019) 7, no. 4, 10.1128/microbiolspec.GPP3-0053-2018. PMC6629438 31298205

[12] Banla L. I. , Salzman N. H. , and Kristich C. J. , Colonization of the Mammalian Intestinal Tract by Enterococci, Current Opinion in Microbiology. (2019) 47, 26–31, 10.1016/j.mib.2018.10.005, 30439685. 30439685 PMC6511500

[13] Comerlato C. B. , Resende M. C. , Caierão J. , and d′Azevedo P. A. , Presence of Virulence Factors in *Enterococcus faecalis* and *Enterococcus faecium* Susceptible and Resistant to Vancomycin, Memórias do Instituto Oswaldo Cruz. (2013) 108, no. 5, 590–595, 10.1590/S0074-02762013000500009. 23903974 PMC3970601

[14] Gholizadeh P. , Köse Ş. , Dao S. , Ganbarov K. , Tanomand A. , Dal T. , Aghazadeh M. , Ghotaslou R. , Ahangarzadeh Rezaee M. , Yousefi B. , and Samadi Kafil H. , How CRISPR-Cas System Could Be Used to Combat Antimicrobial Resistance, Infection and Drug Resistance. (2020) 13, 1111–1121, 10.2147/IDR.S247271, 32368102. 32368102 PMC7182461

[15] Rodrigues M. , McBride S. W. , Hullahalli K. , Palmer K. L. , and Duerkop B. A. , Conjugative Delivery of CRISPR-Cas9 for the Selective Depletion of Antibiotic-Resistant Enterococci, Antimicrobial Agents and Chemotherapy. (2019) 63, no. 11, 10.1128/AAC.01454-19, 31527030. PMC6811441 31527030

[16] Palmer K. L. and Gilmore M. S. , Multidrug-Resistant Enterococci Lack CRISPR-Cas, mBio. (2010) 1, no. 4, 10-1128, 10.1128/mbio.00227-10. PMC2975353 21060735

[17] Tao S. , Chen H. , Li N. , and Liang W. , The Application of the CRISPR-Cas System in Antibiotic Resistance, Infection and Drug Resistance. (2022) Volume 15, 4155–4168, 10.2147/IDR.S370869, 35942309. 35942309 PMC9356603

[18] Murray B. E. , The Life and Times of the Enterococcus, Clinical Microbiology Reviews. (1990) 3, no. 1, 46–65, 10.1128/CMR.3.1.46, 2404568. 2404568 PMC358140

[19] Dutka-Malen S. , Evers S. , and Courvalin P. , Detection of Glycopeptide Resistance Genotypes and Identification to the Species Level of Clinically Relevant Enterococci by PCR, Journal of Clinical Microbiology. (1995) 33, no. 1, 24–27, 10.1128/jcm.33.1.24-27.1995, 7699051. 7699051 PMC227872

[20] Chow J. W. , Aminoglycoside Resistance in Enterococci, Clinical Infectious Diseases. (2000) 31, no. 2, 586–589, 10.1086/313949. 10987725

[21] Martineau F. , Picard F. J. , Grenier L. , Roy P. H. , Ouellette M. , and Bergeron M. G. , Multiplex PCR Assays for the Detection of Clinically Relevant Antibiotic Resistance Genes in Staphylococci Isolated From Patients Infected After Cardiac Surgery, Journal of Antimicrobial Chemotherapy. (2000) 46, no. 4, 527–534, 10.1093/jac/46.4.527, 11020248. 11020248

[22] CLSI , Clinical and Laboratory Standards Institute. Antimicrobial Susceptibility Testing M100, 33rd ed. 2023.

[23] Taji A. , Heidari H. , Shahini-Shamsabadi M. , and Motamedifar M. , High-Level Resistance to Aminoglycosides Among Multidrug Resistant Non-*faecalis* and Non-*faecium* Enterococci, Clinical Laboratory. (2022) 68, no. 9, 10.7754/Clin.Lab.2022.220222, 36125162. 36125162

[24] Norouzi H. , Zandi H. , Madadizadeh F. , and Heidari H. , Comparison of Factors Contributing to Drug Resistance and Infection Severity in *Pseudomonas aeruginosa* Isolates Among COVID-19 and Non-COVID-19 Patients, Molecular Biology Reports. (2025) 52, no. 1, 10.1007/s11033-025-10711-z, 40549117. 40549117

[25] Martín-Platero A. M. , Valdivia E. , Maqueda M. , and Martínez-Bueno M. , Characterization and Safety Evaluation of Enterococci Isolated From Spanish Goats′ Milk Cheeses, International Journal of Food Microbiology. (2009) 132, no. 1, 24–32, 10.1016/j.ijfoodmicro.2009.03.010, 19375810. 19375810

[26] Shahi F. , Hamidi H. , Khoshnood S. , Mehdipour G. , Dezfouli A. A. , and Sheikh A. F. , Virulence Determinants and Biofilm Formation in Clinical Isolates of Enterococcus: A Cross-Sectional Study, Journal of Acute Disease. (2020) 9, no. 1, 27–32, 10.4103/2221-6189.276079.

[27] Jafarzadeh Samani R. , Tajbakhsh E. , Momtaz H. , and Kabiri Samani M. , Prevalence of Virulence Genes and Antibiotic Resistance Pattern in *Enterococcus faecalis* Isolated From Urinary Tract Infection in Shahrekord, Iran, Reports of Biochemistry & Molecular Biology. (2021) 10, no. 1, 50–59, 10.52547/rbmb.10.1.50, 34277868. 34277868 PMC8279714

[28] Shahveh M. , Tajbakhsh E. , Momtaz H. , and Ranjbar R. , Antimicrobial Resistance, Biofilm Formation and Virulence Factors in Enterococcus faecalis Strains Isolated From Urinary Tract Infection in Kermanshah, Iran, Archives of Pharmacy Practice. (2020) 11, no. 3-2020, 79–88.

[29] Ma X. , Zhang F. , Bai B. , Lin Z. , Xu G. , Chen Z. , Sun X. , Zheng J. , Deng Q. , and Yu Z. , Linezolid Resistance in *Enterococcus faecalis* Associated With Urinary Tract Infections of Patients in a Tertiary Hospitals in China: Resistance Mechanisms, Virulence, and Risk Factors, Frontiers in Public Health. (2021) 9, 570650, 10.3389/fpubh.2021.570650, 33614576. 33614576 PMC7893085

[30] Guan L. , Beig M. , Wang L. , Navidifar T. , Moradi S. , Motallebi Tabaei F. , Teymouri Z. , Abedi Moghadam M. , and Sedighi M. , Global Status of Antimicrobial Resistance in Clinical *Enterococcus faecalis* Isolates: Systematic Review and Meta-Analysis, Annals of Clinical Microbiology and Antimicrobials. (2024) 23, no. 1, 10.1186/s12941-024-00728-w, 39182092. PMC11344933 39182092

[31] Zalipour M. , Esfahani B. N. , and Havaei S. A. , Phenotypic and Genotypic Characterization of Glycopeptide, Aminoglycoside and Macrolide Resistance Among Clinical Isolates of *Enterococcus faecalis*: A Multicenter Based Study, BMC Research Notes. (2019) 12, no. 1. 10.1186/s13104-019-4339-4 PMC6537152 31133071

[32] Taji A. , Heidari H. , Ebrahim-Saraie H. S. , Sarvari J. , and Motamedifar M. , High Prevalence of Vancomycin and High-Level Gentamicin Resistance in *Enterococcus faecalis* Isolates, Acta Microbiologica et Immunologica Hungarica. (2019) 66, no. 2, 203–217, 10.1556/030.65.2018.046, 30465449. 30465449

[33] Chouchani C. , El Salabi A. , Marrakchi R. , Ferchichi L. , and Walsh T. R. , First Report of mefA and msrA/msrB Multidrug Efflux Pumps Associated With blaTEM-1 *β*-Lactamase in *Enterococcus faecalis* , International Journal of Infectious Diseases. (2012) 16, no. 2, e104–e109, 10.1016/j.ijid.2011.09.024, 22137270. 22137270

[34] Yang J. X. , Liu C. W. , Wu F. W. , Zhu L. , and Liang G. W. , Molecular Characterization and Biofilm Formation Ability of *Enterococcus faecium* and *Enterococcus faecalis* Bloodstream Isolates From a Chinese Tertiary Hospital in Beijing, International Microbiology. (2024) 27, no. 3, 929–939, 10.1007/s10123-023-00441-2, 37932582. 37932582 PMC11144123

[35] Khdir K. H. , Molecular and Bacteriological Study of *Enterococcus faecalis* Isolated From Different Clinical Sources, Zanco Journal of Pure and Applied Sciences. (2020) 32, no. 2, 157–166.

[36] Hashem Y. A. , Abdelrahman K. A. , and Aziz R. K. , Phenotype-Genotype Correlations and Distribution of Key Virulence Factors in *Enterococcus faecalis* Isolated From Patients With Urinary Tract Infections, Infection and Drug Resistance. (2021) 14, 1713–1723, 10.2147/IDR.S305167, 34007190. 34007190 PMC8123086

[37] Grande R. , Puca V. , and Muraro R. , Antibiotic Resistance and Bacterial Biofilm, Expert Opinion on Therapeutic Patents. (2020) 30, no. 12, 897–900. 32985275 10.1080/13543776.2020.1830060

[38] Kiruthiga A. , Padmavathy K. , Shabana P. , Naveenkumar V. , Gnanadesikan S. , and Malaiyan J. , Improved Detection of esp, hyl, asa1, gelE, cylA Virulence Genes Among Clinical Isolates of Enterococci, BMC Res Notes. (2020) 13, no. 1, 10.1186/s13104-020-05018-0, 32197635. PMC7085142 32197635

[39] Khalil M. A. , Alorabi J. A. , Al-Otaibi L. M. , Ali S. S. , and Elsilk S. E. , Antibiotic Resistance and Biofilm Formation in Enterococcus spp. Isolated From Urinary Tract Infections, Pathogens. (2023) 12, no. 1, 10.3390/pathogens12010034, 36678381. PMC9863506 36678381

[40] Nallapareddy S. R. , Singh K. V. , Sillanpää J. , Zhao M. , and Murray B. E. , Relative Contributions of Ebp Pili and the Collagen Adhesin Ace to Host Extracellular Matrix Protein Adherence and Experimental Urinary Tract Infection by *Enterococcus faecalis* OG1RF, Infection and Immunity. (2011) 79, no. 7, 2901–2910, 10.1128/IAI.00038-11, 21505082. 21505082 PMC3191960

[41] Tendolkar P. M. , Baghdayan A. S. , Gilmore M. S. , and Shankar N. , Enterococcal Surface Protein, Esp, Enhances Biofilm Formation by *Enterococcus faecalis* , Infection and Immunity. (2004) 72, no. 10, 6032–6039, 10.1128/IAI.72.10.6032-6039.2004, 15385507. 15385507 PMC517584

[42] Zheng J. X. , Wu Y. , Lin Z. W. , Pu Z. Y. , Yao W. M. , Chen Z. , Li D. Y. , Deng Q. W. , Qu D. , and Yu Z. J. , Characteristics of and Virulence Factors Associated With Biofilm Formation in Clinical *Enterococcus faecalis* Isolates in China, Frontiers in Microbiology. (2017) 8, 10.3389/fmicb.2017.02338, 29225595. PMC5705541 29225595

[43] Tao S. , Chen H. , Li N. , Fang Y. , Xu Y. , and Liang W. , Association of CRISPR-Cas System With the Antibiotic Resistance and Virulence Genes in Nosocomial Isolates of *Enterococcus* , Infection and Drug Resistance. (2022) 15, 6939–6949, 10.2147/IDR.S388354, 36474907. 36474907 PMC9719680

[44] Tao S. , Zhou D. , Chen H. , Li N. , Zheng L. , Fang Y. , Xu Y. , Jiang Q. , and Liang W. , Analysis of Genetic Structure and Function of Clustered Regularly Interspaced Short Palindromic Repeats Loci in 110 *Enterococcus* Strains, Frontiers in Microbiology. (2023) 14, 1177841, 10.3389/fmicb.2023.1177841, 37168121. 37168121 PMC10165109

[45] Huo W. , Price V. J. , Sharifi A. , Zhang M. Q. , and Palmer K. L. , *Enterococcus faecalis* Strains With Compromised CRISPR-Cas Defense Emerge Under Antibiotic Selection for a CRISPR-Targeted Plasmid, Applied and Environmental Microbiology. (2023) 89, no. 6, e0012423, 10.1128/aem.00124-23, 37278656. 37278656 PMC10304774

[46] Gholizadeh P. , Aghazadeh M. , Ghotaslou R. , Rezaee M. A. , Pirzadeh T. , Cui L. , Watanabe S. , Feizi H. , Kadkhoda H. , and Kafil H. S. , Role of CRISPR-Cas system on Antibiotic Resistance Patterns of *Enterococcus faecalis* , Annals of Clinical Microbiology and Antimicrobials. (2021) 20, no. 1, 10.1186/s12941-021-00455-6, 34321002. PMC8317297 34321002

[47] Price V. J. , McBride S. W. , Hullahalli K. , Chatterjee A. , Duerkop B. A. , and Palmer K. L. , Enterococcus faecalis CRISPR-Cas Is a Robust Barrier to Conjugative Antibiotic Resistance Dissemination in the Murine Intestine, mSphere.(2019) 4, no. 4, 10-1128, 10.1128/mSphere.00464-19. PMC6656873 31341074

[48] Costache C. , Colosi I. , Toc D. A. , Daian K. , Damacus D. , Botan A. , Toc A. , Pana A. G. , Panaitescu P. , Neculicioiu V. , Schiopu P. , Iordache D. , and Butiuc-Keul A. , CRISPR-Cas System, Antimicrobial Resistance, and *Enterococcus* Genus-A Complicated Relationship, Biomedicines. (2024) 12, no. 7, 10.3390/biomedicines12071625, 39062198. PMC11274382 39062198

[49] Pandova M. , Kizheva Y. , and Hristova P. , Relationship Between CRISPR-Cas Systems and Acquisition of Tetracycline Resistance in Non-Clinical *Enterococcus* Populations in Bulgaria, Antibiotics. (2025) 14, no. 2, 10.3390/antibiotics14020145, 40001389. PMC11852239 40001389

[50] Hullahalli K. , Rodrigues M. , and Palmer K. L. , Exploiting CRISPR-Cas to Manipulate *Enterococcus faecalis* Populations, Elife. (2017) 6, 10.7554/eLife.26664. PMC5491264 28644125

[51] Lindenstrauss A. G. , Pavlovic M. , Bringmann A. , Behr J. , Ehrmann M. A. , and Vogel R. F. , Comparison of Genotypic and Phenotypic Cluster Analyses of Virulence Determinants and Possible Role of CRISPR Elements Towards Their Incidence in *Enterococcus faecalis* and *Enterococcus faecium* , Systematic and Applied Microbiology. (2011) 34, no. 8, 553–560, 10.1016/j.syapm.2011.05.002, 21943678. 21943678

[52] Zhao T. , Fan J. , Abu-Zaid A. , Burley S. K. , and Zheng X. F. S. , Nuclear mTOR Signaling Orchestrates Transcriptional Programs Underlying Cellular Growth and Metabolism, Cells. (2024) 13, no. 9, 10.3390/cells13090781, 38727317. PMC11083943 38727317

[53] Fan J. , Yuan Z. , Burley S. K. , Libutti S. K. , and Zheng X. F. S. , Amino Acids Control Blood Glucose Levels Through mTOR Signaling, European Journal of Cell Biology. (2022) 101, no. 3, 151240, 10.1016/j.ejcb.2022.151240, 35623230. 35623230 PMC10035058

[54] Fan J. , Khanzada Z. , and Xu Y. , Mechanisms Underlying Muscle-Related Diseases and Aging: Insights Into Pathophysiology and Therapeutic Strategies, Muscles. (2025) 4, no. 3, 10.3390/muscles4030026, 40843913. PMC12371960 40843913

[55] Motallebi M. , Seyyedi Z. S. , and Azadchehr M. J. , Genetic Diversity, Antimicrobial Resistance, and Virulence Factors of *Enterococcus faecalis* Isolates Obtained From Stool Samples of Hospitalized Patients, Jundishapur Journal of Microbiology. (2022) 15, no. 6, 1–11, 10.5812/jjm-121379.

[56] Alnakshabandi W. M. , Merza N. S. , Khaled H. M. , and Jubrael J. M. , Study Genetic Relationship Among *Enterococcus faecalis* Strains Collected From Urine Harbored Different Virulence Profiles Using ERIC-PCR Assay, Journal of Duhok University. (2020) 23, no. 1, 63–68, 10.26682/sjuod.2020.23.1.7.

